# Efficient Non-Epigenetic Activation of HIV Latency through the T-Cell Receptor Signalosome

**DOI:** 10.3390/v12080868

**Published:** 2020-08-08

**Authors:** Joseph Hokello, Adhikarimayum Lakhikumar Sharma, Mudit Tyagi

**Affiliations:** 1 Department of Basic Science, Faculty of Science and Technology, Kampala International University-Western Campus, P.O Box 71, Bushenyi, Uganda; hokello.joseph@kiu.ac.ug; 2 Center for Translational Medicine, Thomas Jefferson University, 1020 Locust Street, Philadelphia, PA 19107, USA; LakhikumarSharma.Adhikarimayum@jefferson.edu

**Keywords:** HIV, non-epigenetics, transcription factors, latency, reactivation

## Abstract

Human immunodeficiency virus type-1 (HIV-1) can either undergo a lytic pathway to cause productive systemic infections or enter a latent state in which the integrated provirus remains transcriptionally silent for decades. The ability to latently infect T-cells enables HIV-1 to establish persistent infections in resting memory CD4+ T-lymphocytes which become reactivated following the disruption or cessation of intensive drug therapy. The maintenance of viral latency occurs through epigenetic and non-epigenetic mechanisms. Epigenetic mechanisms of HIV latency regulation involve the deacetylation and methylation of histone proteins within nucleosome 1 (nuc-1) at the viral long terminal repeats (LTR) such that the inhibition of histone deacetyltransferase and histone lysine methyltransferase activities, respectively, reactivates HIV from latency. Non-epigenetic mechanisms involve the nuclear restriction of critical cellular transcription factors such as nuclear factor-kappa beta (NF-κB) or nuclear factor of activated T-cells (NFAT) which activate transcription from the viral LTR, limiting the nuclear levels of the viral transcription transactivator protein Tat and its cellular co-factor positive transcription elongation factor b (P-TEFb), which together regulate HIV transcriptional elongation. In this article, we review how T-cell receptor (TCR) activation efficiently induces NF-κB, NFAT, and activator protein 1 (AP-1) transcription factors through multiple signal pathways and how these factors efficiently regulate HIV LTR transcription through the non-epigenetic mechanism. We further discuss how elongation factor P-TEFb, induced through an extracellular signal-regulated kinase (ERK)-dependent mechanism, regulates HIV transcriptional elongation before new Tat is synthesized and the role of AP-1 in the modulation of HIV transcriptional elongation through functional synergy with NF-κB. Furthermore, we discuss how TCR signaling induces critical post-translational modifications of the cyclin-dependent kinase 9 (CDK9) subunit of P-TEFb which enhances interactions between P-TEFb and the viral Tat protein and the resultant enhancement of HIV transcriptional elongation.

## 1. Introduction

With the introduction of the highly active antiretroviral therapy (HAART), the morbidity and mortality rate among HIV-infected individuals has reduced dramatically [1]. HAART, by virtue of restricting human immunodeficiency virus (HIV) levels, greatly enhances the host immune system [2]. The continuous use of HAART reduces the viremia in plasma beyond detection levels [3,4,5]. Nevertheless, using sensitive methods, residual viremia may still be detected in some individuals, validating the inability of HAART in eradicating HIV [6]. The ability of HIV to establish latent infections at the level of individual T-cells remains the main barrier to eradicate HIV [7]. The latent reservoir begins to establish quickly, within a few days of the initial infection [8]. The most-stable reservoir of HIV resides in the transcriptionally-silent resting memory CD4+ T-cells [9,10]. In order to express its genes, HIV requires host cell transcription machinery. Therefore, in transcriptionally-inert resting memory T-cells, HIV latency is established due to the unavailability of transcription factors [11]. Although the amount of latently infected cells is very limited (approximately one in one million of resting T-cells), a highly stable pool of latently-infected cells are always present in HIV patients, which remains a barrier for human immunodeficiency virus type-1 (HIV-1) eradication in patients undergoing effective HAART therapy [12]. The latent reservoir maintains its capacity to produce an infectious virus upon the discontinuation of the antiretroviral therapy (ART) and reactivation of harboring cells by recall antigens or various cytokines [13,14]. In this review, we discuss how the T-cell receptor (TCR) activation efficiently induces nuclear factor-kappa beta (NF-κB), nuclear factor of activated T-cells (NFAT), and activator protein 1 (AP-1) transcription factors and how these factors efficiently regulate HIV long terminal repeats (LTR) transcription either individually or through functional synergy with one another through the non-epigenetic mechanism. Furthermore, we will shed light on post-translational modifications of the cyclin-dependent kinase 9 (CDK9) subunit of positive transcription elongation factor b (P-TEFb) which enhances the interactions between P-TEFb and the viral Tat protein and the resultant enhancement of HIV transcriptional elongation.

## 2. The T-Cell Receptor Signalosome

**Figure 1:**
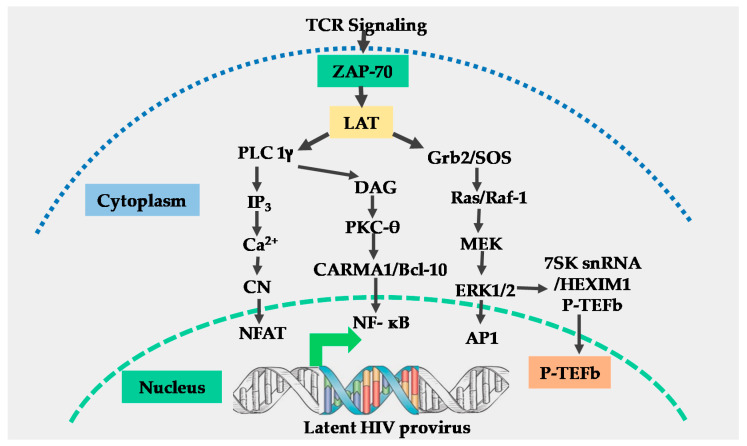
Schematic model for T-cell receptor signaling. Engagement of the T-cell receptor (TCR)/CD3 complex by the peptide-MHC complex requires the stabilization of the TCR-peptide/MHC complex interaction by CD4 or CD8 molecules, resulting in the activation of the p56 leukocyte kinase (Lck) which phosphorylates the immunoreceptor tyrosine-based activation motifs (ITAMS) within the cytoplasmic tails of CD3-zeta (not shown), resulting in the generation and transmission of the TCR signals. Engagement of the CD28 co-receptor (not shown) present on the surface of T-cells by B7 ligands on antigen-presenting cells is required for full TCR activation. Following full TCR activation, the zeta associated protein 70 (ZAP-70) and linker of activated T-cells (LAT) are activated. Activation of ZAP-70 and LAT results in the induction of three main signal pathways, namely: the calcium–calcineurin pathway which induces nuclear factor of activated T-cells (NFAT); the protein kinase C pathway which induces nuclear factor-kappa beta (NF-κB) and the MAP kinase/ extracellular signal-regulated kinase (ERK) pathway which induces activator protein 1 (AP-1) and positive transcription elongation factor b (P-TEFb). All these transcription factors translocate into the nucleus to regulate human immunodeficiency virus (HIV) transcription.

The T-cell receptor (TCR) is a multi-complex structure comprised of polymorphic protein molecules on the cell surface that interact with the peptide-MHC complex and non-polymorphic transmembrane CD3-zeta molecules, which transmit signals resulting from the TCR engagement and antigen recognition leading to T-cell activation [15,16]. The immediate outcome of the TCR/CD3 ligation by the peptide-MHC complex is the rapid phosphorylation of immunoreceptor tyrosine-based activation motifs (ITAMS) on the cytoplasmic tail of the TCR/CD3 complex by the Src family protein tyrosine kinase called leukocyte kinase-56 (Lck56) [15,17]. The phosphorylation of ITAMS leads to the recruitment of cytosolic tyrosine kinase, zeta-associated protein-70 (ZAP-70) to the plasma membrane where it phosphorylates multiple transmembrane adapter molecules including the T-lymphocyte-specific linker for activated T-cells (LAT) at multiple tyrosine residues [15,17,18]. Phosphorylated LAT recruits several multi-protein scaffold molecules including Grb-2, phospholipase C-gamma (PLC-g), and phosphoinositide-3 kinase (PI3K (Figure 1)).

Grb-2 interacts with guanine nucleotide exchange factor SOS to activate the Ras-mitogen-activated protein kinase (MAPK) signal transduction pathway which leads to the induction of activator protein-1 (AP-1) [19] and P-TEFb through an ERK-dependent mechanism [20]. The hydrolysis of phosphatidylinositol 4, 5 bisphosphates (PIP-2) by PLC-gamma generates two-second messenger molecules; inositol 1, 4, 5 triphosphate (IP3) and diacylglycerol (DAG). The interaction of IP3 with its receptor at the cell membrane leads to intracellular Ca2+ mobilization resulting in activation of the calcium–calcineurin signal pathway that induces NFAT transcription factors (Figure 1) [15,18].

Through the canonical pathway of NF-κB induction, DAG activates the protein kinase C (PKC) pathway resulting in NF-κB mobilization via the CARMA1, Bcl110, and MALT1 (CBM) protein complex present upstream of the IκB-α kinase (IKK) complex [15,17,18]. Phosphatidylinositide-3 kinase enhances PKC-mediated NF-κB induction by activating Akt kinase which synergizes with the PKC activation pathway [21,22]. The efficient activation of the TCR/CD3 complex by the peptide-MHC complex ligation requires a co-stimulatory signal. Several families of co-stimulatory proteins are present on antigen-presenting cells (APCs) that interact with their receptors within the immunological synapse during the peptide-MHC presentation to T-lymphocytes [16,22]. The TCR-mediated antigen-specific stimulation is essential for initiating T-cell activation, signaling through the TCR has to be regulated through co-stimulatory or co-inhibitory receptors, which are important for full TCR activation or suppression of the T-cell responses [23]. The most important and well-studied co-stimulatory signal required for full TCR/CD3 activation is the B7 on APCs which, comprises CD80 and CD86 molecules. B7 interacts with CD28 molecules which are constitutively expressed on T-lymphocytes to enhance NF-κB induction resulting from TCR activation signals [16,22,24]. Other than the B7 co-stimulatory molecule, other co-stimulatory molecules that enhance the TCR signals have also been reported. For instance, Barat and Tremblay [25] demonstrated that the co-stimulation of CD43 along with the TCR activation augmented the nuclear mobilization of NF-κB and NFAT transcription factors independent of the CD28 co-stimulation. The augmented nuclear induction of NF-κB and NFAT enhanced HIV LTR transcription. In a different set of experiments, Tardif and Tremblay [26] demonstrated that CD81 also provides a co-stimulatory signal to the TCR signals which increased HIV LTR gene expression. On the other hand, the CTLA-4 and PD-1 are known to mediate the inhibitory functions in T-cell activation and signaling, and both are expressed only after cellular activation where they function in the feedback inhibition of T-cell activation. The PD-1 inhibits TCR activation by inducing the dephosphorylation of the TCR upstream signaling molecules through the transient recruitment of the phosphatase SHP2 while CTLA-4 competes with CD28 for CD80 and CD86 binding [27].

It is imperative to note that as much as independent signaling pathways usually lead to the induction of specific transcription factors, there is significant crosstalk between the TCR signaling pathways especially upstream of the kinases and phosphatases that activate the specific transcription factors. For instance, the LcK56, which is required for canonical TCR signaling leading to T-cell responses, also mediates a negative feedback loop through the phosphatase SHP2 that turns off the TCR signaling [28]. On the other hand, the HIV-1 protein nef has been reported to modulate the TCR activation signals to promote HIV replication and pathogenesis. For instance, Fenard et al. [29] reported that HIV nef increases the expression of the TCR-induced transcription factors NF-κB and NFAT following TCR activation. They reported that this was achieved through nef-induced priming of the TCR signaling pathways which occurs at a proximal step before protein kinase C (PKC) activation. Similarly, Neri et al. [30] also demonstrated that the modulation of TCR signaling by the HIV-1 nef results in a superinduction of NFAT transcription factor and interleukin 2 (IL-2) production. The superinduction of NFAT was shown to favor HIV-1 replication in both quiescent and metabolically active CD4 T-cells. These observations were confirmed by Fortin et al. [31]. Therefore, other than the inherent TCR modulation, the HIV nef protein can also modulate the TCR signaling pathways in order to enhance HIV transcription and pathogenesis. 

## 3. The NF-κB Transcription Factors

The NF-κBs are members of the superfamily of DNA binding transcription factors that regulate several important biological functions including innate and adaptive immune responses [32,33]. The NF-κB (or Rel) transcription factors consist of five members all of which contain the conserved N-terminal Rel homology domain (RHD (~ 300 amino acids)). The RHD harbors both the DNA binding and the NF-κB dimerization domains. The nuclear localization sequence (NLS) is also found within the RHD. Unlike p50 and p52, which lack the transcription activation domain (TAD), p65 (or RelA), RelB and c-Rel contain the TAD and, for this reason, p50 and p52 are unable to activate transcription [34]. The NF-κB proteins can form functional hetero- or homodimers within its members. Heterodimers of p65 and p50 are the most abundant and transcriptionally active forms, although homodimers of p65 are also known to potently activate transcription [34]. Heterodimers of p50 and p52 or their homodimers are unable to activate transcription because p50 and p52 lack the TAD. The RHD comprises two independently folded immunoglobulin-like sub-domains called RHR-N and RHR-C. Rel homology region N is used in the recognition and binding of specific DNA sequences while RHR-C mediates NF-κB dimerization and binding to the IκB-α inhibitor [32,35]. The IκBs are a small family of related proteins containing ankyrin repeats at the N-terminus and a PEST motif at the C-terminus. The major form and the best studied IκBs is the IκB-α, which binds NF-κB dimers and stearically blocks its nuclear localization sequence (NLS), thereby, causing its sequestration in the cytoplasm [32]. However, following the activation of the canonical pathway of NF-κB induction through the TCR or stimulation with pro-inflammatory cytokines such as TNF-α, the catalytic subunit IKK-β, together with the regulatory subunit IKK-γ (also known as NEMO) becomes activated and phosphorylates IκB-α, leading to its ubiquitination, the proteasomal degradation and nuclear localization of NF-κB to regulate the transcription of NF-κB-responsive genes [32].

## 4. The NFAT Transcription Factors

The NFAT proteins are another member of the superfamily of transcription factors with similar properties to those of the NF-κB. For instance, both NFAT, and NF-κB rapidly translocate into the nucleus upon TCR activation; they both bind to similar DNA sequences and both are known to form functional synergy with other transcription factors such as those belonging to the bZIP class of transcription factors [33]. However, while NF-κB modulates both innate and adaptive immune responses, NFAT, which is a T-lymphocyte-specific transcription factor, regulates T-lymphocyte activation, proliferation, and differentiation, a process required for adaptive immune responses. 

**Figure 2:**
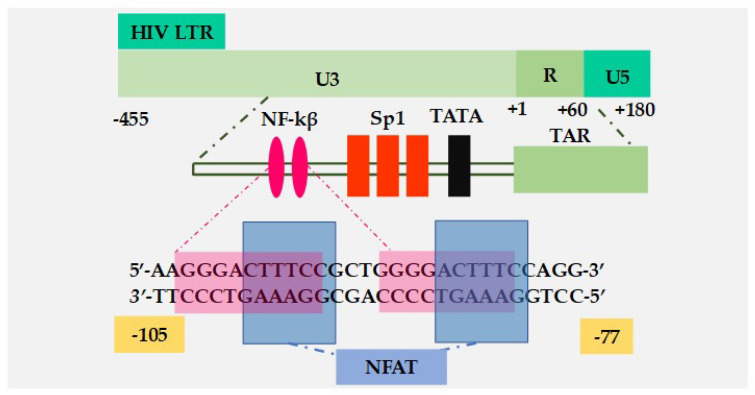
Structure of the HIV long terminal repeats (LTR) showing overlapping NF-κB and NFAT binding sites. The HIV core promoter within the LTR harbors two NF-κB and three Sp1 binding sites and a TATA-box from which HIV transcription initiates. However, NFAT binding sites overlap the two NF-κB binding sites. Both NF-κB and NFAT have unique binding sequence requirements and the binding of NF-κB and NFAT to the LTR to regulate HIV-1 transcription is mutually exclusive.

Members of the NFAT family are comprised of five NFAT proteins, namely NFAT1 (also known as NFATc2 or NFATp) [36,37], NFAT2 (NFATc1 or NFATc) [36], NFAT3 (NFATc4) [38], NFAT4 (NFATc3 or NFATx), and NFAT5 [39,40]. The expression of NFAT proteins are cell-type specific. Whereas NFATc2 is constitutively expressed in numerous lymphoid cell types, NFATc1 is predominantly expressed in T-lymphocytes [41]. Following the initial exposure of naïve T-lymphocytes to antigens, two isoforms of NFATc1, namely isoform B (NFATc1/B) and isoform C (NFATc1/C), are synthesized for the first time. However, during T-cell differentiation into effector T-lymphocytes, activated T-lymphocytes then acquire the potential to rapidly induce the synthesis of NFATc1 isoform A (NFATc1/A) following subsequent exposure to antigen or mitogenic stimulation [41]. The expression of the NFATc1 isoform is due to a differential usage of the proximal splice/poly-A site. In naïve T-lymphocytes, the proximal splice/poly-A site required for NFATc1/A expression is silent. However, during T-lymphocyte differentiation, to become effector T-lymphocytes, the concentration of polyadenylation factor CstF-64 significantly increases resulting in an inefficient recognition of the proximal splice/poly-A site which regulates NFATc1/A expression [41]. Unlike NFATc1/A, which has an expression that is activation-induced upon subsequent TCR activation, NFATc1/B and NFATc1/C expression in T-lymphocytes are constitutive. In unactivated T-lymphocytes, inactive, heavily phosphorylated NFAT proteins, except NFAT5 [39,40], are sequestered in the cytoplasm. Following TCR engagement [42], the calcium–calmodulin-dependent calcineurin phosphatase dephosphorylates NFAT proteins, resulting in the exposure of their nuclear localization signals thereby leading to its nuclear translocation and the activation of NFAT-responsive promoters. However, NFAT5 is constitutively a nucleophosphoprotein regardless of calcineurin activation and does not synergize with AP-1 transcription factors [39]. Both NFAT and NF-κB share a highly conserved DNA binding RHD and indeed, the two transcription factors bind to similar DNA binding sequences [35] (Figure 2).

In NFATc2, RHR-N also mediates specific DNA binding in addition to mediating NFAT interactions with AP-1 c-Fos and c-Jun transcription factors [13]. On the other hand, RHR-C mediates the homodimeric binding of NFATc2 to the HIV LTR. Like NF-κB, NFAT proteins also have the transcription activation domain (TAD) which recruits other transcription regulatory proteins to activate transcription [43].

## 5. The AP-1 Transcription Factors

Activator protein-1, induced by mitogen-activated protein kinase (MAPK) signaling of the TCR, is yet another group of transcription factors known to regulate the HIV provirus transcription [44,45]. The MAPK cascades are a common mechanism of signal transduction in many cellular processes. These serine/threonine kinases are present in many cell types and cooperate in transmitting extracellular signals, which regulate a variety of biological functions including cellular proliferation, differentiation, development, stress response, and apoptosis [46]. To date, four distinct MAPK cascade pathways have been elucidated and named according to the subgroup of their respective MAPK components, namely: (a) the extracellular signal-regulated protein kinase-1 and 2 (ERK1/2); (b) the c-Jun N-terminal protein kinase/stress-activated protein kinase (JNK/SAPK); (c) the p38-MAPK [46,47] and (d) the ERK5 cascade also known as the big MAPK (BMK) [46].

In addition to the TCR activation, the MAPK signaling is activated by a variety of stimuli such as mitogen, growth factors, and cytokines [48]. The distinct MAPK cascades appear to be specific in their physiological response. ERK1/2 is activated by extracellular stimuli such as growth factors, hormones, and neurotransmitters, and plays a role in cellular proliferation and differentiation [46]. Any extracellular agent that acts through the G-protein coupled receptors, tyrosine kinase receptors or ion channels can initiate a variety of intracellular signaling responses that results in the activation of the ERK1/2 cascade.

The JNK/SAPK and the p38-MAPK cascades respond mainly to stress and are involved in stress responses and apoptosis. On the other hand, the ERK5/BMK cascade transmits both mitogen-activated and stress-induced signals [46]. However, the functions of the different cascades may also be cell type-dependent. For instance, ERK1/2 may participate in the apoptotic response under a particular condition, whereas the JNK/SAPK may coordinate cell survival and proliferation [46]. Although other putative MAPK family proteins such as ERK7 and ERK8 have been identified, they may not be activated by the extracellular stimuli that typically activate ERK1/2 and they do not respond to common activation signals that induce JNK/SAPK or p38-MAPK signaling [46]. The ERKs are ubiquitous serine/threonine kinases, which phosphorylate and activate a wide range of substrates including AP-1 transcription factors. The AP-1 proteins are nucleophosphoproteins belonging to the bZIP class of transcription factors. The AP-1 transcription factors consist of either c-Jun homodimers or the c-Fos/c-Jun heterodimers. The Jun family proteins include c-Jun, JunB, and JunD while the Fos protein family members include the c-Fos, FosB, Fra-1, and Fra-2 [44]. Unlike c-Jun, which forms functional homo- and heterodimers, c-Fos proteins are only active as heterodimers with members of the Jun proteins and on their own are unable to activate AP-1-dependent transcription except when they synergize with other transcription factors, such as NFAT or NF-κB [49].

## 6. Influence of TCR Activation on HIV Infection

Peterlin and colleagues [50] were one of the first groups to determine a role for TCR signaling in the activation of HIV LTR transcription. Because of their quiescent nature, naïve CD4+ T-cells are unable to support productive HIV infection. Therefore, efficient HIV infection and replication occur in activated CD4+ T-cells [9], meaning that TCR activation is required to sustain the HIV infection and replication. This is due to the fact that, unlike naïve CD4+ T-cells, activated CD4+ T-cells contain plenty of metabolites such as nucleotides and amino acids that are required for virus transcription and protein expression. However, upon fulfillment of their effector functions, the activated CD4+ T-cells revert back to become quiescent resting memory CD4+ T-cells. Following the process of reversion back into quiescence, some of the quiescent resting memory CD4 T-cells that were infected as effector CD4+ T-cells then harbor the latent HIV proviruses, and this is the process through which HIV latency is established and maintained [7]. Subsequently, the reactivation of quiescent resting memory CD4+ T-cells following the encounter with the same antigen results in the activation of latent HIV proviruses from memory CD4+ T-cells due to the mobilization of the TCR-induced transcription factors and their translocation into the nucleus following the TCR activation. One of the hallmarks of HIV infections is the profound depletion of the CD4+ T-cells marked by Th17 T-cells dysfunction at the mucosal level. However, HAART restores the Th1 T-cells subset, but not the Th17 T-cells subset. In a genome-wide transcriptional profiling in memory CD4+ T-cells enriched in Th17 cells, Cleret Buhot et al. [51] identified a set of HIV dependency cellular factors, which are typically TCR signal molecules in Th17 cells versus Th1 cells, which could potentially be targeted as novel therapies aimed at protecting Th17 cells from HIV infections and the subsequent depletion in HIV-infected individuals. Although the induction of HIV transcription usually occurs in cells latently infected with HIV following TCR activation, not all latently infected T-cells respond to TCR stimulation. To this effect, Rezaei et al. [52] observed that latency can be established through multiple ways, and as such the pathway through which HIV latency is established is critical to how it is maintained and reversed. Therefore, TCR activation is a requirement not only for the reactivation of HIV latency, but also for the efficient HIV infection and replication within CD4+ T-cells. 

## 7. Molecular Control of HIV Gene Expression

The transcription factors that are discussed above, including NF-κB, NFAT, and AP-1, are present in the cytoplasm. However, following the activation of the TCR, these factors are mobilized and translocate into the nucleus where they significantly contribute to the regulation of HIV gene expression. The HIV LTR contains *cis*-regulatory elements that specifically bind these factors. The integrated HIV provirus serves as the template for the synthesis of viral mRNAs which encode a full complement of viral structural, regulatory, and accessory proteins required for virus replication and virulence [53,54]. The HIV-1 LTR is the site for transcription initiation and harbors multiple cis-regulatory elements required for virus mRNA synthesis. The LTR is made up of three distinct regions, namely the unique 3′ (U3) end, repeated (R) sequence, and unique 5′ (U5) end. The U3 comprises elements that mediate RNAP II binding to the viral template DNA including the TATA-box located at the −28 nucleotide position upstream of the transcription start site (+1). Two NF-κB and three sp1 binding sites are located at the 5′ end of the TATA-box [53,54] (Figure 2).

The initiation of HIV-1 transcription from the LTR begins with the binding of a highly conserved 38KD TATA-box binding protein (TBP) to the TATA-box sequence. Binding of the TBP initiates the recruitment of additional factors to form the pre-initiation complex referred to as the TBP-associated factors (TAF). The multi-protein complex formed between TBP and TAF is referred to as TFIID and it is the minimal protein complex that induces basal transcription from the HIV-1 LTR. However, the efficient initiation of transcription from the LTR requires the interaction of upstream enhancer binding factors such as NF-κB or NFAT with TFIID [54,55]. Transcription factor II H (TFIIH) phosphorylates the C-terminal domain (CTD) of RNAP II within the initiation complex to enhance transcription elongation [54,56,57]. However, in the absence of the viral transcription transactivator protein Tat, HIV-1 transcription is less efficient and RNAP II stalls a few nucleotides from the transcription start site following promoter clearance.

In the presence of the viral Tat protein, the synthesis of viral mRNA is greatly enhanced due to increased elongation efficiency mediated by Tat resulting in the generation of singly-spliced and unspliced full-length viral RNA transcripts [58,59,60,61]. Tat acts through binding unusually to an RNA element known as transactivation response element (TAR), a stem-loop structure formed at the 5′ end of nascent viral RNA transcripts following transcription through the first 59 nucleotides [54]. The binding of Tat to TAR leads to the recruitment of positive transcription elongation factor b (P-TEFb), a complex comprising of cyclin T1 and cyclin-dependent kinase 9 (CDK9), the kinase subunit, which hyperphosphorylates the CTD of the largest subunit of RNAP II leading to enhanced RNAP II processivity and transcription efficiency [55].

### 7.1. Role of NF-κB in HIV-1 Transcription

Two NF-κB binding sites are located at the HIV promoter (Figure 2) and the binding of NF-κB to this sequence induces HIV transcriptional initiation. Due to basal transcription, latently infected CD4^+^ T-lymphocytes contain sub-threshold levels of the viral Tat protein. However, NF-κB mobilization into the nucleus from the cytoplasm initiates the synthesis of new viral Tat which binds to the TAR element, an RNA stem-loop structure formed at the 5′ end of viral RNA in synergy with the cellular transcription elongation factor P-TEFb [62]. The CDK9 subunit of P-TEFb then phosphorylates the c-terminal domain (CTD) of the largest subunit of RNA polymerase II (RNAP II), resulting in enhanced HIV-1 transcriptional elongation [62]. The presence of Tat in the transcription system increases the provirus transcription elongation efficiency by about 100-fold. Using a clone 2D10 model system of HIV-1 latency—where HIV-1 Tat is expressed in *cis* and d2EGFP replaces nef to monitor the shutdown kinetics of HIV-1 during entry into latency and reactivation from latency— Pearson and colleagues [63] demonstrated that indeed, the activation of latent HIV-1 is strictly dependent on NF-κB and viral Tat.

### 7.2. Role of NFAT in HIV-1 Transcription

Initial studies implicating NFAT as a positive regulator of HIV-1 transcription were performed by Kinoshita and colleagues [64,65]. Given the tight correlation of HIV-1 transcription to T-lymphocyte activation, Kinoshita et al., through transfection assays, demonstrated that NFATc1 activates HIV-1 transcription in SupT1 cell lines by binding to sequences within the NF-κB binding sites (nucleotide −104 to −81 (Figure 2)). Though DNA footprinting, Kinoshita and his group further demonstrated that although NFATc1 enhances the HIV-1 transcriptional response through binding to the NF-κB regulatory element on the HIV-1 LTR, there are specific sequence requirements for NFATc1 binding to this region, which are independent of the residues comprising the canonical NF-κB binding sequence, an observation that was later confirmed by Cron and colleagues [66]. In addition to the NFAT binding sequence that overlaps the two NF-κB binding sites within the HIV LTR, putative NFAT binding sites are present upstream of the NF-κB sites, although Markovitz and colleagues demonstrated that these upstream NFAT *cis*-regulatory elements have no regulatory effects on HIV-1 transcription [67].

In an attempt to determine the role of NFAT in the regulation of HIV transcription, Cron et al., transfected primary human CD4+ T lymphocytes with LTR-luciferase reporter plasmids containing mutant NF-κB binding sites along with a plasmid expressing recombinant NFATc1 or NFATc2 [66]. Cron and colleagues demonstrated that NFATc1 or NFATc2 substantially increased p24/Gag antigen expression following the stimulation of transfected cells with ionomycin alone or in combination with phorbol esters. Furthermore, cross-linking of the TCR/CD3 complex using anti-CD3 and anti-CD28 mAbs also increased LTR activity, which was significantly inhibited by cyclosporine A (CsA) or FK506, which are immunosuppressive drugs both of which inhibit the activation-induced nuclear translocation of NFAT proteins. The abolition of HIV LTR transcriptional activity demonstrates the importance of NFAT in the activation of HIV transcriptional responses consistent with recent reports that NFAT is the major regulator of HIV transcription in primary CD4+ T-lymphocytes [68].

Consistent with the findings of Kinoshita and colleagues, Cron et al. also demonstrated that the activation of HIV transcription by NFAT or NF-κB has distinct motif requirements. The *cis*-acting elements of NFAT on the HIV LTR promoter overlap the duplicate NF-κB binding sites and include a 4 bp spacer nucleotides (−93 to −90) that separate the two NF-κB binding sites (Figure 2). Mutation of these 4 bp spacer nucleotides (GCTG to ATAT) sufficiently blocks NFAT binding without significant effects on the ability of the HIV promoter to respond to the activation signals mediated by NF-κB [62,66]. Although both NFAT and NF-κB recognize overlapping yet distinct DNA binding sequences within the LTR, these two transcription factors bind mutually exclusively to the HIV LTR to regulate HIV transcription following TCR activation.

### 7.3. Regulation of HIV-1 Transcription by MAPK-Activated AP-1 

Multiple AP-1 binding sites have been identified on the HIV-1 LTR promoter. Roebuck and colleagues [69] reported that AP-1 binds to phorbol ester-responsive elements located within the 5′ noncoding region of the HIV LTR known as the downstream sequence element (DSE) located at nucleotide positions +84 to +105 and +149 to +170 to modulate HIV-1 transcription. On the other hand, Van Lint and colleagues [70] previously showed that there are three intragenic AP-1 binding sequences within the *pol* gene located at nucleotide positions +4079 to +4342 in the HXB2 HIV molecular clone. When the intragenic AP-1 enhancer elements were cloned upstream of the thymidine kinase promoter and transfected into HeLa cells, these sequences functioned as 12-O-tetradecanoylphorbol-13-acetate (TPA)-inducible enhancers. Gel mobility assays and competition experiments using these intragenic AP-1 enhancer sequences demonstrated that two out of three AP-1 binding sites firmly bound affinity-purified AP-1 proteins or AP-1 obtained from TPA-induced HeLa nuclear extracts. Van Lint and colleagues further showed that there were three additional AP-1 sites downstream of the HIV transcription start site located at nucleotide positions +465 to +720 and an AP-3-like motif, which when mutated resulted in defective HIV-1 replication. Franza and colleagues [71] also reported the presence of AP-1 sites upstream of the two NF-κB binding sites within the LTR.

Despite the existence of multiple AP-1 sites on the HIV LTR promoter region, Yang et al. [72] demonstrated that the critical regulation of HIV-1 transcriptional activity by AP-1 appears to require a functional interaction of AP-1 with transcription factors associated with the NF-κB binding sequences on the HIV-1 LTR. Using transient transfection and reporter gene assays in U1 and U937 cell lines derived from human monocyte and macrophage lineages, respectively, Yang and colleagues demonstrated that AP-1 physically and functionally interacts with NF-κB, thereby forming a transcription ternary complex that synergistically transactivates HIV-1 gene expression [72]. Using different plasmid constructs—pLTR-Luc (WT LTR), pLTRs-Luc (LTR derived from WT pLTR-Luc but with deleted sequences upstream of the NF-κB binding sites) and pLTRmκB (LTR with mutant NF-κB binding sites derived from WT pLTR-Luc)—Yang and colleagues demonstrated that it is the functional interaction between AP-1 and NF-κB not the upstream AP-1 sites on the HIV LTR or the AP-1 sites downstream of the transcription start site or the AP-1 sites within the pol gene that were required for the regulation of HIV-1 transcriptional activity by AP-1 [72]. In a yeast two-hybrid system and by gel mobility assay, they also demonstrated the physical interactions of NF-κB with AP-1 c-Fos and c-Jun proteins. Consistent with the work of Yang and colleagues, Greene and his group demonstrated that physical interactions between AP-1 and NF-κB are mediated via the bZIP and RHD regions, respectively, and that functional synergy required an active TAD of both AP-1 and NF-κB [73].

Indeed, Hokello et al., (in communication) demonstrated that TCR activation is complex and results in the induction of multiple transcription factors via distinct signal pathways including NF-κB, NFAT, and AP-1 transcription factors with unique nuclear induction kinetics and nuclear levels. In a related experiment, Kim et al. [56] demonstrated that TCR activation using anti-CD3 and anti-CD28 mAbs results in the nuclear mobilization of active pools of P-TEFb through an ERK-dependent mechanism, a critical co-factor for the HIV Tat protein, which enhances HIV-1 transcriptional elongation during the early activation time points before new Tat is synthesized. Taken together, these observations demonstrate the critical contribution of AP-1 transcription factors acting with NF-κB or NFAT through functional synergy in enhancing the efficiency of the HIV transcriptional response following TCR activation. Similarly, in 2013, Mbonye et al. [74] tested the hypothesis that TCR activation and signaling induce critical post-translational modifications which result in enhanced interactions between P-TEFb and viral Tat proteins. To their amazement, they observed that TCR activation strongly induced the phosphorylation of the CDK9 subunit of P-TEFb at serine-175 (Ser175), and this modification of CDK9 strengthened CDK9 and Tat intermolecular interactions. It is imperative to note here that the CDK9 subunit of P-TEFb recruits the HIV Tat protein to regulate HIV transcriptional elongation. Mbonye et al. concluded that CDK9 phosphorylation at Ser175 is critical in altering the cooperative binding of Tat and bromodomain-4 (BRD4) to P-TEFb and that phosphorylation of CDK9 at Ser175 is a molecular marker for transcriptionally active forms of P-TEFb.

## 8. Role of HIV LTR Sequence Diversity on HIV Transcriptional Response

The genetic analysis of the HIV LTR sequences of viral subtypes circulating in India revealed subtype-specific variability in several HIV LTR regulatory sequences [75]. Similarly, Neogi et al. [76] analyzed the diversity of HIV-1 LTR sequences following mother-to-child HIV transmission in North India. They observed that HIV-1 subtype C LTR harbored three NF-κB binding sites while subtype B harbored two NF-κB binding sites. However, an analysis of intra-subtype sequence divergence between subtypes B and C revealed a greater sequence diversity in subtype B LTR sequences than subtype C. Significant sequence divergence for both subtypes B and C was found in NFAT and USF transcription factors binding sites within the HIV LTR, while the NF-κB, Sp-1, Ets-1, AP-1, and TATA-box binding sequences were highly conserved in both HIV subtypes analyzed. Furthermore, de Arellano et al. [77] analyzed and compared the HIV LTR sequences including the regulatory, enhancer, promoter and TAR regions in 59 HIV-1-infected individuals known to be infected with non-B HIV subtypes and found out that up to 34% of the samples analyzed were LTR recombinants. Further, the LTR sequences displayed a high degree of genetic variability among distinct HIV-1 subtypes with subtype-specific markers which could potentially influence the interactions with cellular transcription factors resulting in differential transcriptional responses among distinct HIV-1 clades. Indeed, in a separate set of experiments, de Arellano et al. [78] reported a drastic decrease in HIV transcriptional responses due to a hypermutation of the HIV LTRs in different viral subtypes and recombinants. A similar observation in the HIV LTR subtype-specific transcriptional response was also reported by Jeeninga and colleagues [79]. In consideration of the fact that the *cis*-regulatory sequences within the HIV LTR between different HIV subtypes are highly variable, it is conceivable that these HIV LTR sequences’ diversity significantly impacts on the role and contribution of transcription factors that are induced following the TCR activation in the reactivation and regulation of HIV LTR transcription.

## 9. Conclusions and Prospects for Novel Drug Discovery

Human immunodeficiency viruses can either undergo a lytic growth pathway to produce new virions or enter a latent state in which the integrated HIV provirus remains transcriptionally silent for decades. Despite strong humoral and cellular immune responses against the viral proteins, the ability to latently infect individual T-cells enables HIV-1 to establish persistent infections in a small proportion of memory CD4+ T-lymphocytes. To date, no chemotherapy with 100% efficacy is available for HIV therapy due to its ability to establish latent infections, which are capable of reactivating productive lytic infections upon the cessation or disruption of highly active antiretroviral therapy (HAART), nor is there a vaccine to control HIV infection. The current HAART regime utilizes antiretroviral drugs that target viral proteins such as protease and integrase which are essential for virus replication. This approach presents major limitations and is prone to drug resistance development due to high mutation rates associated with the low fidelity of HIV transcription.

There is, therefore, an urgent need for new therapeutic approaches to tackle not only the problem of viral drug resistance that is so rampant with the current HAART regime, but also the bigger problem of HIV latency which is the greatest obstacle to current efforts to effectively treat and cure HIV infection. The molecular mechanism of the regulation of HIV transcription following TCR activation provides a great opportunity for the discovery of a novel class of antiviral drugs with a unique mechanism of action that would reactivate latent HIV proviruses in the latent pool, and also drug-resistant viral variants from the latently infected cellular reservoirs.

Conceivably, the use of such a novel class of antiviral drugs and small molecules, which reactivate HIV from latency and results in the depletion of the latent HIV provirus pools in combination with the current HAART regime, could potentially lead to effective HIV treatment and its eradication. Currently, despite the presence of small molecules that target specific TCR signal pathways to induce specific transcription factors, the currently available small molecules have produced mixed results with a variety of off-target effects due to a number of reasons including (1) a lack of high specificity and potency in primary cells, (2) crosstalk in the TCR signaling pathways and (3) the inability to induce all the critical factors, including the P-TEFb, which are vital in the efficient reactivation and regulation of HIV transcription. These limitations, therefore, indicate the dire need to discovering new small molecules which will require novel high throughput screening techniques in the appropriate cellular backgrounds. It is, however, imperative to note that any such approach to reactivate latent provirus pools using small molecules should be able to induce P-TEFb, a cellular co-factor for viral Tat that regulates HIV transcriptional elongation. Most noticeably, TCR signals are known to activate P-TEFb through an ERK-dependent mechanism and, therefore, small molecules that can intracellularly and selectively activate specific TCR signal pathways such as MAPK/ERK in combination with PKC pathways can potentially be employed without causing toxic global TCR activation. Such an approach would enable the selective induction of AP-1, NF-κB, and P-TEFb transcription factors to purge out the latent provirus pools, but not NFAT which is responsible for global T-cell activation.
